# Supplementary material for: Protective Factors in the LGBTIQ+ Adolescent Experience: A Systematic Review
Source: Healthcare (Basel). 2024 Sep 16;12(18):1865. doi: 10.3390/healthcare12181865 (PMC11431086; doi:10.3390/healthcare12181865)
Supplement: Supplementary file 1 [file healthcare-12-01865-s001.zip › healthcare-3124346-supplementary.pdf]

Table S1. Mixed methods. Quantitative data.

Studies	Question/objective sufficiently described	Study design evident and appropiate	Method of subject/comparision group selection or souce of information/input variables described and appropriate	Subject (and comparision group) characteristics sufficiently described	Outcome and applicable exposure measure(s) well defined and robust to measurement/misclassification bias? Means of assessment reported?	Sample size approppiate?	Analytic methods described / justified and appropriate?	Some stimate of variance is reported for the main results?	Controlled for confounding?	Results reported in sufficient detail?	Conclusions supported by results?	Total	Weighted total
Wilhelm et al., (2023) [7]	2	2	1	2	1	2	2	2	2	2	2	20	0.91
Pariseau et al., (2019) [33]	2	2	2	1	2	1	2	2	Not applicable	2	2	18	0.90
Luthar et al., (2021) [34]	2	2	1	1	2	1	2	2	1	2	2	18	0.82
Cardona et al., (2023) [35]	2	2	1	1	1	1	2	2	Not applicable	2	2	16	0.80
Andrzejewski et al., (2023) [36]	2	2	2	2	2	2	2	2	2	2	2	22	1.00

Table S2. The critical appraisal tool. Qualitative data.

Studies	Purpose of study	Research design				Sampling					Data collection			Data analysis			Findings		Value of research		Trust worthiness and reliability	
	Q.1	*Q. 2*	*Q. 3*	*Q. 4*	*Q. 5*	*Q. 6*	*Q. 7*	*Q. 8*	*Q. 9*	*Q. 10*	Q. 11	*Q. 12*	*Q. 13*	*Q. 14*	*Q. 15*	*Q. 16*	*Q. 17*	*Q. 18*	*Q. 19*	*Q. 20*	*Q. 21*	*Q. 22*
Morgan et al (2022) [37]	Yes	Yes	Yes	No	Yes	Yes	Yes	Yes	Yes	Yes	Yes	Yes	Yes	Yes	Yes	Yes	Yes	Yes	Yes	Yes	Yes	Yes
Johnson et al., (2020) [38]	Yes	Yes	Yes	Yes	Yes	Yes	Yes	Yes	Yes	Yes	Yes	Yes	Yes	Yes	No	Yes	Yes	Yes	Yes	Yes	Yes	Yes
Pampati et al., 2021) [39]	Yes	Yes	Yes	Yes	Yes	No	Yes	Yes	Yes	Yes	Yes	Yes	Yes	Yes	No	Yes	Yes	Yes	Yes	Yes	Yes	Yes
Bridge et al., (2022) [40]	Yes	Yes	Yes	Yes	Yes	Yes	Yes	Yes	Yes	Yes	Yes	Yes	Yes	Yes	No	Yes	Yes	Yes	Yes	Yes	Yes	Yes
Miller et al., (2022) [20]	Yes	Yes	Yes	Yes	Yes	Yes	Yes	Yes	Yes	Yes	Yes	Yes	Yes	Yes	Yes	Yes	Yes	Yes	Yes	Yes	Yes	Yes
Greenfield et al., (2021) [41]	Yes	Yes	Yes	No	Yes	No	Yes	Yes	Yes	Yes	Yes	No	Yes	Yes	Yes	Yes	Yes	Yes	Yes	Yes	Yes	Yes

Table S3. Quantitative methods.

Studies	Question/objective sufficiently described	Study design evident and appropiate	Method of subject/comparision group selection or souce of information/input variables described and appropriate	Subject (and comparision group) characteristics sufficiently described	Outcome and applicable exposure measure(s) well defined and robust to measurement/missclassification bias? Means of assessment reported?	Sample size appropppiate?	Analytic methods described/ justified and appropriate?	Some stimate of variance is reported for the main results?	Controlled for confounding?	Results reported in sufficient detail?	Conclusions supported by results?	Total	Weighted total
Greenspan et al., (2023) [65]	2	2	2	2	2	1	2	2	Not applicable	2	2	19	0.95
Vanbronkhorst et al., (2021) [12]	2	2	2	2	2	2	2	2	2	2	2	22	1.00
Rivas-Koehl et al., (2021) [73]	2	2	2	2	2	2	2	1	Not applicable	2	2	19	0.95
Whitton et al., (2021) [48]	2	2	2	2	2	2	2	2	Not applicable	2	2	20	1.00
Lardier et al., (2020) [49]	2	2	2	2	2	2	2	2	Not applicable	2	2	20	1.00
Chan & Leung (2023) [61]	2	2	2	2	2	2	2	2	Not applicable	2	2	20	1.00

Garthe et al., (2020) [50]	2	2	2	1	2	1	2	1	Not applicabl e	2	2	17	0.85
Hong et al., (2021) [51]	2	2	2	1	2	1	1	2	2	2	2	19	0.86
Rider et al., (2022) [5]	2	2	2	2	2	1	1	0	2	1	2	17	0.77
Stroem et al., (2021) [52]	2	2	1	1	2	2	2	2	2	2	2	20	0.91
Valido et al., (2022) [3]	2	2	1	2	2	2	1	1	1	1	2	17	0.77
Hatchel et al., (2019) [42]	2	2	1	2	2	2	2	2	2	2	2	21	0.95
Valido et al., (2021) [69]	2	2	1	2	2	2	2	1	2	2	2	20	0.91
Ross-Reed et al., (2019) [53]	2	2	1	2	2	1	1	2	1	2	2	18	0.82
Berona et al., (2021) [54]	2	2	1	2	2	1	2	2	Not applicabl e	1	1	16	0.80
Hong et al.., (2023) [51]	2	2	2	2	2	1	1	2	2	1	2	19	0.86
Julian et al., (2021) [43]	1	2	2	2	2	1	2	2	0	2	2	18	0.82

Chinazzo et al., (2023) [44]	2	2	2	2	2	1	2	2	Not applicable	2	2	19	0.95
Conn et al., (2023) [64]	2	1	2	2	2	2	2	2	Not applicable	2	2	19	0.95
Speer et al., (2022) [81]*	1	1	2	2	2	1	1	0	0	1	2	13	0.59
Horwitz et al., (2021) [55]	2	2	2	2	2	1	1	2	1	2	2	19	0.86
Mintz et al., (2021) [72]	1	2	1	2	2	2	2	0	1	2	2	17	0.77
Wang et al., (2023) [56]	2	2	1	2	1	1	2	0	2	1	2	16	0.73
Taliaferro et al., (2019) [71]	2	2	2	2	2	2	2	2	Not applicable	2	2	20	1.00
Semprevivo (2023) [57]	2	2	2	2	2	2	2	2	2	2	2	22	1.00
Taliaferro et al., (2019) [58]	2	2	2	2	2	2	2	2	2	2	2	22	1.00
Pollitt et al., (2021) [59]	1	2	2	2	2	1	2	2	Not applicable	2	1	17	0.85
Eisenberg et al.,	1	2	2	2	1	1	2	2	2	1	2	18	0.82

(2021) [68]													
Parmar et al., (2022) [63]	1	2	2	2	1	1	2	2	2	1	2	18	0.82
Jadva et al., (2023) [45]	1	2	1	2	2	2	1	2	Not applicable	1	2	16	0.80
Ioverno (2023) [62]	2	2	2	2	2	2	1	2	Not applicable	2	2	19	0.95
McPherson et al., (2023) [66]	2	2	1	2	2	2	2	2	2	1	2	20	0.91
Eisenberg et al., (2019) [46]	2	2	2	2	2	2	1	0	Not applicable	1	1	15	0.75
McKay and Watson (2020) [60]	2	2	2	2	2	2	2	0	Not applicable	2	2	18	0.90
Standley and Foster-Fishman (2021) [82]*	2	2	1	1	2	1	2	2	0	0	1	14	0.64
Zell & Kerr (2024) [67]	2	2	2	2	2	1	2	2	2	2	2	21	0.95

Burstein et al., (2023) [70]	1	2	2	2	2	1	2	0	2	2	2	18	0.82

***New References for supplemental material**

[81] Speer, S. R.; Atteberry-Ash, B.; Kattari, S. K.; Kattari, L.; Gupta, R.; Walls, N. E. An Intersectional Modeling of Risk for Nonsuicidal Self-Injury among LGBTQ Adolescents. J Child Fam Stud, **2022**, 31 (4), 1158–1171. https://doi.org/10.1007/s10826-022-02250-z.

[82] Standley, C. J.; Foster-Fishman, P. Intersectionality, Social Support, and Youth Suicidality: A Socioecological Approach to Prevention. In Suicide and Life-Threatening Behavior; John Wiley and Sons Inc, **2021**; Vol. 51, pp 203–211. https://doi.org/10.1111/sltb.12695.
